# Melatonin Preserves the Postharvest Quality of Cut Roses through Enhancing the Antioxidant System

**DOI:** 10.3390/plants11202713

**Published:** 2022-10-14

**Authors:** Ragia M. Mazrou, Sabry Hassan, Mei Yang, Fahmy A.S. Hassan

**Affiliations:** 1 Horticulture Department, Faculty of Agriculture, Menoufia University, Shebin El Kom 32516, Egypt; 2 Department of Biology, College of Science, Taif University, P.O. Box 11099, Taif 21944, Saudi Arabia; 3 College of Forestry, Guangxi University, Nanning 530004, China; 4 Horticulture Department, Faculty of Agriculture, Tanta University, Tanta 31527, Egypt

**Keywords:** antioxidant enzymes, glutathione, gene expression, lipid peroxidation, membrane stability, vase life

## Abstract

The vase life of cut rose is relatively short, therefore; preserving its postharvest quality via eco-friendly approaches is of particular economic importance. From the previous literature, despite melatonin (MT) plays diverse important roles in the postharvest quality maintenance, its impact on preserving the postharvest quality of cut flowers is really scarce. This research therefore was undertaken to find out the possibility of exogenous MT as an eco-friendly preservative to extend the vase life of cut roses. The flowering stems of *Rosa hybrida* cv. ‘First Red’ were pulsed in MT solutions at 0, 0.1, 0.2 and 0.3 mM for 30 min and then transferred to distilled water for evaluation. The vase life was significantly prolonged and relative water content was considerably maintained due to MT application compared to the control, more so with 0.2 mM concentration which nearly doubled the vase life (1.9-fold) higher than the control. SEM investigation showed that MT treatment reduced the stomatal aperture in lower epidermis which was widely opened in control flowers. MT treatment significantly increased the phenol content, glutathione (GSH) content and CAT, APX and GR enzyme activities compared to untreated flowers. Additionally, the radical scavenging capacity in MT-treated flowers was considerably higher than that of control and therefore MT treatment reduced H_2_O_2_ production and lipid peroxidation, which altogether reflected in membrane stability maintenance.

## 1. Introduction

Rose (*Rosa hybrida* L.) is a flower with important economic value and it is the major exportable cut flower crop worldwide [1]. Rose is called Queen or King of Flowers and there is no any cut flower surpasses it for its colour, fragrance and beauty. 

The rapid postharvest physiological deterioration and inducing senescence, however, largely reduce its quality and marketability and therefore limit its commercial value [2]. Therefore, how to keep the quality of postharvest cut roses has always been a concern of several investigators. Rose flower senescence is characterized by limiting water supply to the flowers that cause water relation interruption and decrease the vase life [3]. Another factor which causes quality deterioration during cut flower handling is oxidative stress which is accelerated by metabolic processes occurring naturally after cutting from the mother plant [4]. Furthermore, flower stem cutting itself causes oxidative injury and therefore overproduction of reactive oxygen species (ROS) that attack the cellular proteins, nucleic acids, and membrane lipids leading to membrane deterioration [5]. During postharvest deterioration of cut roses, ROS levels markedly increased, followed by the activities related to the antioxidant system [1]. 

Studies have revealed that the maintenance of a strong antioxidant machinery to scavenge ROS is associated with a longer vase life in several cut flower species [6,7,8,9]. To regulate overproduction of ROS in an unfavorable condition, plants possess an efficient antioxidative defense machinery comprising of both enzymatic and nonenzymatic systems [10]. Otherwise, during flower senescence, ROS homeostasis is disturbed due to activation of ROS generation or attenuating ROS scavenging, resulting in a pronounced increase in ROS [11] which cause oxidative damage to membrane lipids and proteins, further aggravating postharvest senescence progress [9]. In fact, the observation that ROS triggers the flower senescence progression has been documented by the increment in ROS production in senescent gladiolus and rose cut flowers [1,6]. Alleviating the oxidative damage is therefore a vital issue in preserving the flower quality and extending the vase life. 

Recently, the effect of several compounds on the antioxidant potential to scavenge ROS has increased. For sustainable production of cut flowers, special interest has received some natural substances as alternatives to common chemicals [12] since they have been reported as environmentally friendly and do not compromise the human health. Some of these natural compounds are salicylic acid, volatile oils, chitosan and moringa leaf as well as seed extracts, have been described as ROS scavengers and thus, delaying senescence process and extending the vase life [1,5,6,9,13]. Nevertheless, some of these substances are not commercially reasonable due to low customer preference or need for verifying their effectiveness. In this context, we hypothesize that ROS homeostasis maintenance has a key function in modulating flower senescence of cut roses. Therefore, new and effective methods are required to preserve the quality and extend the vase life of cut roses.

Melatonin (N-acetyl-5-methoxytryptamine, MT), a derivative of tryptophan, is an important small molecular indoleamine hormone that is widely detected in various plant species [14]. In plants, tryptophan is also a substrate source for indole-3-acetic acid (IAA), which means that MT also has a plant hormone-like regulatory impact in plant systems [15]. In this connection, it has been reported that MT is a kind of multifunctional hormone, analogous to IAA in structure [16]. MT, as a new hormone and master regulator, plays a positive role in several physiological processes, such as germination, regulating plant growth and development, flowering, delaying senescence, photosynthesis, postharvest physiology and resisting abiotic stress [17,18,19]. Despite the diverse functions of MT, it is considered as a powerful free radical scavenger and antioxidant, boosting plant performance against oxidative damage [20]. 

Furthermore, MT as a safe and beneficial indoleamine, mitigates the abiotic and biotic stresses via acting as an antioxidant that directly eliminates ROS, activating the antioxidant machinery by upregulating the transcript level and enhancing the enzyme activity, and enhancing the efficiency of other antioxidants [21,22]. Accordingly, MT induces substantial changes in several physiological processes, particularly in multiple stress conditions [18]. The antioxidant capacity of MT has been reported [23] and recent reports have shown that endogenous signaling molecules such as MT could enhance the postharvest quality of several fruits [24]. 

Noticeably, the information about melatonin functions in postharvest quality responses is mainly from vegetables [16,25,26] and fruits [24,27,28], but little is available regarding the roles that MT play in preserving the postharvest quality of cut flowers. In this connection, as far as we know, only two reports [29,30] investigated the effects of exogenous MT on improving the postharvest quality of anthurium and carnation. Indeed, there is no information about the influence of MT on maintaining the postharvest quality of cut rose flowers and its underlying regulatory mechanism. Based on the previous studies, it is speculated that exogenous MT may extend the vase life of cut roses via protecting them from oxidative damage after harvest by triggering its antioxidant system. Therefore, the objective of current study was to investigate the efficacy of exogenous MT on preserving the postharvest quality of cut rose and its underlying physiological and biochemical mechanisms. 

## 2. Results

### 2.1. Vase Life

**Figure 1:**
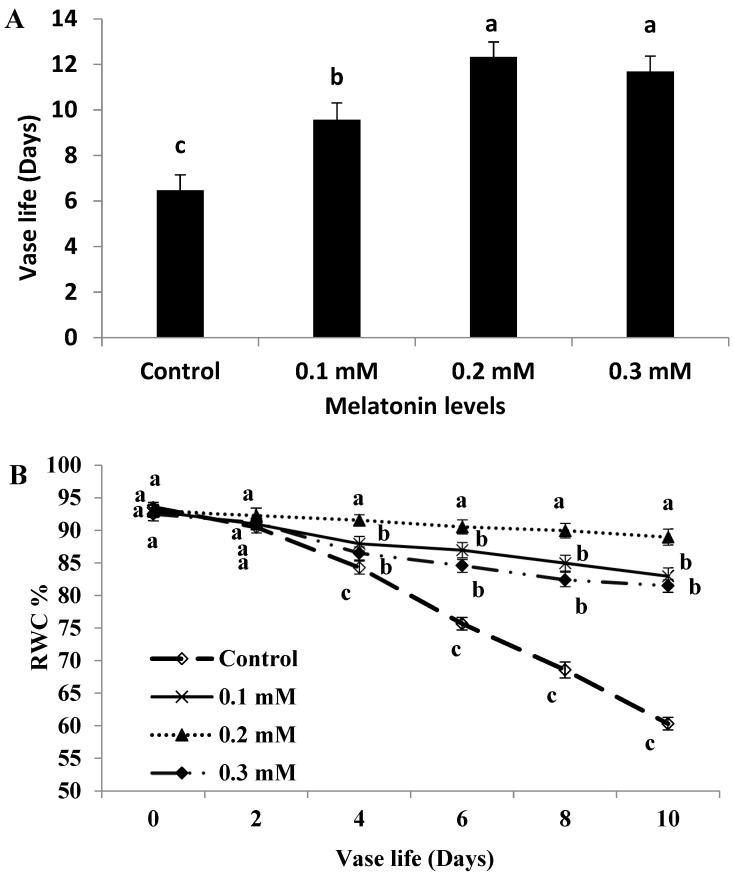
Vase life (**A**) and relative water content (**B**) of cut roses treated with melatonin at 0.1, 0.2 and 0.3 mM. Each value is the mean ± SE of two experiments. Values that have different letters are significantly different based on Tukey-Kramer’s multiple range test at *p* ≤ 0.05.

The vase life of cut roses cv. First Red was significantly (*p* ≤ 0.05) extended due to all MT treatments compared with the control (Figure 1A). The longest flower life (12.33 d) was recorded by 0.2 mM MT-level. Relative to untreated flowers, the exogenous application of MT at 0.2 mM increased the vase life by 90.57% which was nearly double that of the control. Otherwise, the highest MT level (0.3 mM) slightly reduced the flower life in comparison with 0.2 mM level but without significant difference.

### 2.2. Relative Water Content (RWC) 

The RWC was gradually decreased during the vase life period in MT-treated and non-treated flowers and this reduction was clearly observed after the third day of the evaluation period, however; the reduction was significant in non-treated flowers (Figure 1B). Otherwise, MT treatment markedly maintained the RWC and significantly suppressed this reduction compared to the control, more so with 0.2 mM level. A sharp and significant reduction in RWC was detected in untreated flowers from day 2 to day 10.

### 2.3. SEM Investigation of Leaf Stomata

**Figure 2:**
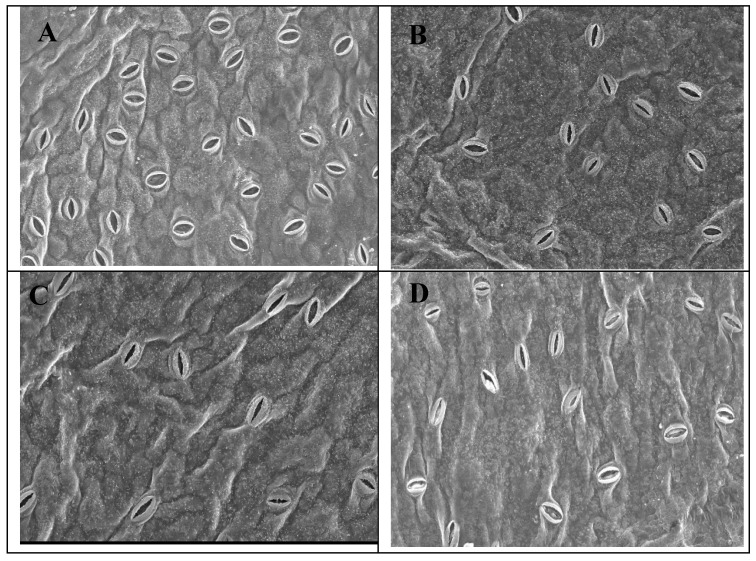
SEM investigations of stomata detected on the lower epidermis of cut *Rosa hybrida* cv. ‘First Red’. Flowers were pulsed for 30 min with distilled water as control (**A**) or melatonin at 0.1, 0.2 and 0.3 mM ((**B**), (**C**) and (**D**), respectively). The investigation was done on day 6 of the vase life period at 300× magnification.

SEM observations on day 6 of stomata found on abaxial leaf surface showed that a large proportion of stomata were widely opened on untreated leaves (Figure 2A) while stomata observed in MT-treated flowers were partially opened (Figure 2B–D). Interestingly, stomata observed on leaves in 0.2 mM MT-treated flowers were more closed compared to those treated with 0.1 or 0.3 mM MT levels.

### 2.4. Total Phenol Content 

**Figure 3:**
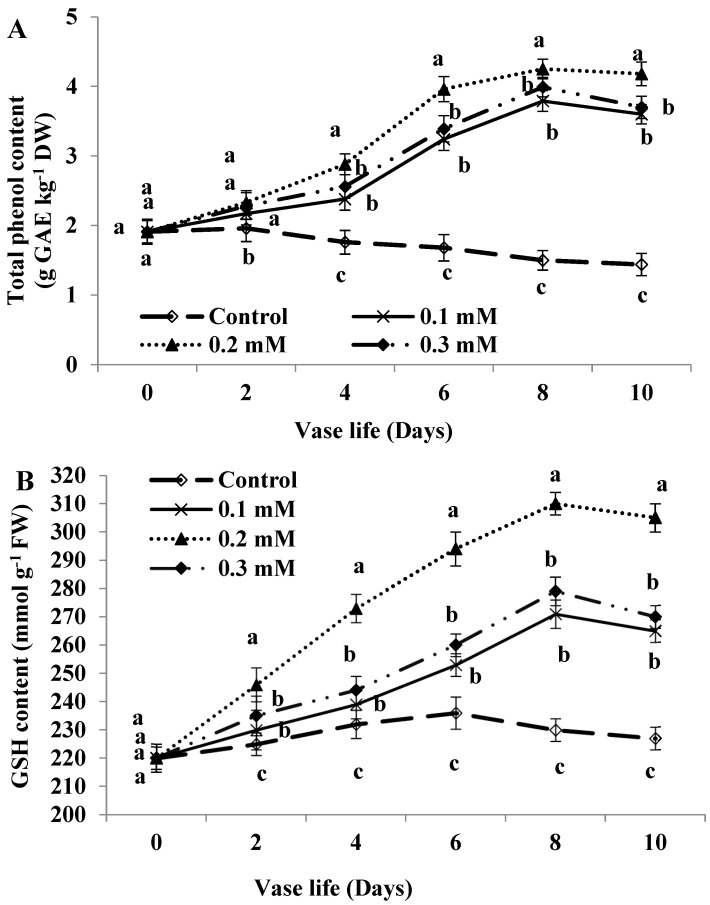
Total phenol content (**A**) and glutathione (GSH) content (**B**) of cut roses treated with melatonin at 0.1, 0.2 and 0.3 mM. Each value is the mean ± SE of two experiments. Values that have different letters are significantly different based on Tukey-Kramer’s multiple range test at *p* ≤ 0.05.

The total phenol content was slightly increased from day 0 to day 2 but it decreased thereafter with the age of flowers (Figure 3A). On the other hand, all MT treatments significantly increased the phenol content during the evaluation period compared to the control, more so with 0.2 mM level. The highest phenol content was observed at day 8 in treated flowers and the differences between 0.1 and 0.3 mM levels were insignificant. The total phenols in MT-treated flowers was increased by 152.66, 183.33 and 166% relative to the control for 0.1, 0.2 and 0.3 mM levels, respectively at day 8.

### 2.5. Glutathione Content 

All MT treatments significantly increased the GSH content in rose flowers compared to untreated control through the evaluation period and reached their maximum values at day 8 (Figure 3B). In control flowers, a slight increase was detected until day 6 and then decreased at the subsequent days. The highest GSH content was observed by MT treatment at 0.1 mM level since this treatment significantly increased the GSH content by 34.78% relative to untreated flowers at day 8.

### 2.6. Antioxidant Enzyme Activity

**Figure 4:**
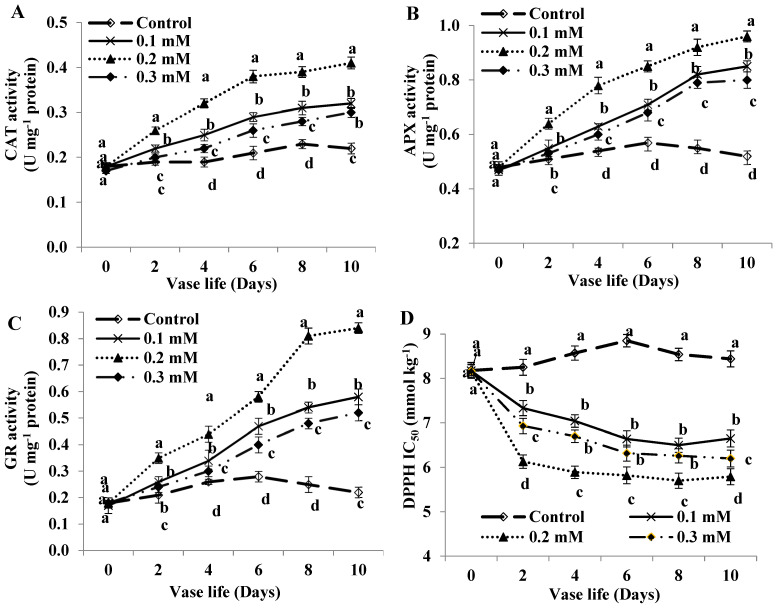
Activity of: (**A**) catalase (CAT); (**B**) Ascorbate peroxidase (APX); (**C**) glutathione reductase (GR) enzyme and (**D**) radical scavenging activity of cut roses treated with melatonin at 0.1, 0.2 and 0.3 mM. Each value is the mean ± SE of two experiments. Values that have different letters are significantly different based on Tukey-Kramer’s multiple range test at *p* ≤ 0.05.

MT treatment resulted in a significant increase in CAT, APX and GR enzyme activities compared to untreated flowers (Figure 4A–C). The activities of these enzymes in control flowers were significantly lower than those in treated ones throughout the evaluation period. A slight increase in antioxidant enzyme activities were observed in untreated flowers until day 6 and then the activity was reduced, however; a great increase in the activities of CAT, APX and GR enzymes were detected in MT-treated flowers until day 10, more so with 0.2 mM level.

### 2.7. Radical Scavenging Activity 

The radical scavenging capacity in MT-treated flowers was significantly increased relative to the control throughout the evaluation period (Figure 4D). The highest scavenging activity (lowest IC_50_, 66.74%) was observed in MT-treated flowers at 0.2 mM level followed by 0.3 and 0.1 mM levels (73.30 and 76.11% of control flowers, respectively at day 8). 

### 2.8. H_2_O_2_ Production and MDA Content 

**Figure 5:**
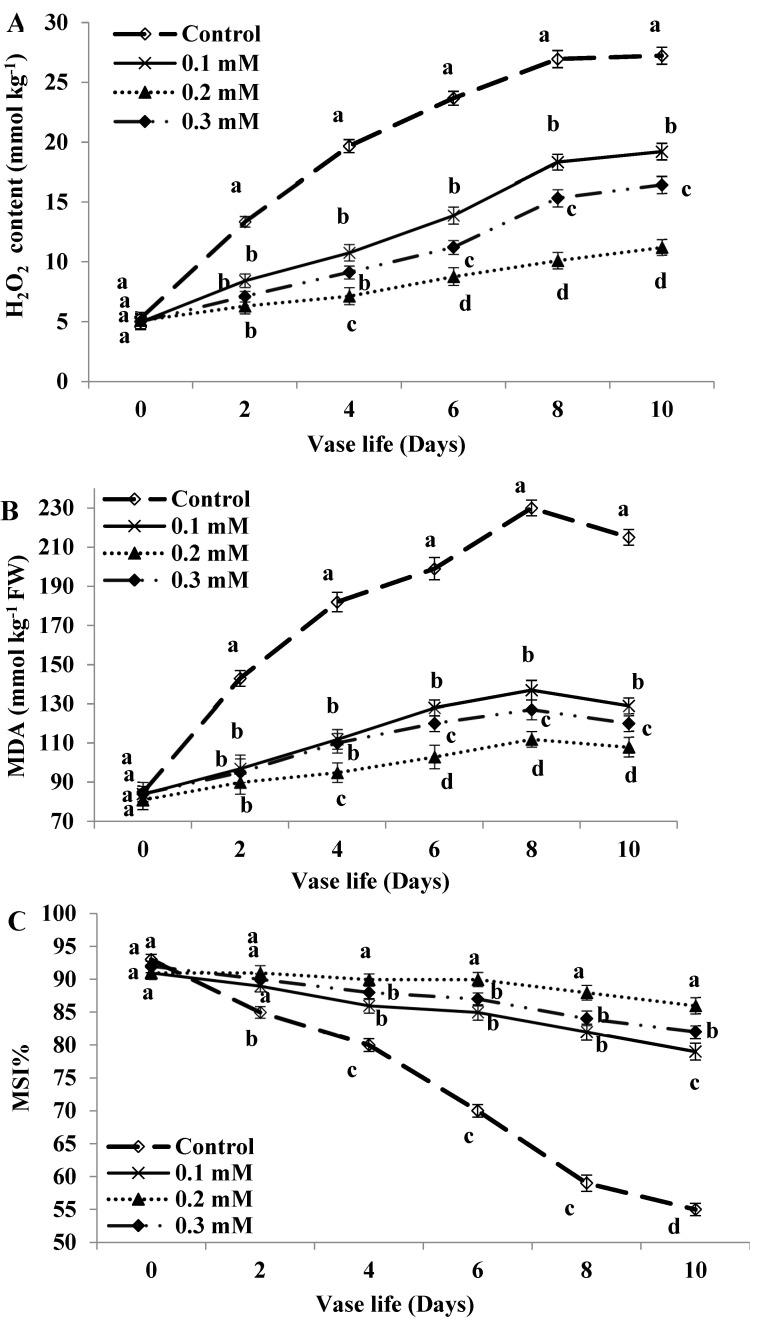
H_2_O_2_ production (**A**) Malondialdehyde content (**B**), and membrane stability index (**C**) of cut roses treated with melatonin at 0.1, 0.2 and 0.3 mM. Each value is the mean ± SE of two experiments. Values that have different letters are significantly different based on Tukey-Kramer’s multiple range test at *p* ≤ 0.05.

The production of H_2_O_2_ was gradually increased in control flowers and reached the peak by day 8 and then decreased at day 10 (Figure 5A). However, MT-treated flowers had significantly decreased H_2_O_2_ production compared to the control, the impact was greatest with 0.2 mM level than 0.1 or 0.3 mM levels. In the same connection, MDA content was gradually increased during the evaluation period of control flowers, the highest value was also observed on day 8. The rise in MDA content was also detected in MT treatment but significantly reduced by all levels compared to the control, more decline in MDA content was reached by 0.2 mM level (Figure 5B). 

### 2.9. Membrane Stability Index (MSI)

Control flowers lost the membrane stability rapidly as shown by a sharp reduction in MSI with the age of cut flowers since it recorded 55% at day 10 (Figure 5C). Otherwise, MT application overcome such adverse effect and maintained the MSI relative to the control, more so with the MT level of 0.2 mM (MSI was 86% versus 79 and 82% with 0.1 and 0.3 mM at day 10, respectively).

## 3. Discussion

Enhancing the productivity and postharvest quality via eco-friendly approaches is of great importance [13,31,32]. This investigation is the first to show the capability of MT to enhance the quality of the postharvest as well as prolonging the cut rose vase life. The mechanisms thereby MT application exhibited its effects were through maintenance of water relations and antioxidant defense systems, which in turn reduced the oxidative damage. Also, MT application modulated stomatal aperture and aquaporin gene expression. It is evident that endogenous MT content impacts the flower senescence and its level, despite flower species, decreases along the flower development and reaches the peak at senescence stage [33]. The vase life extension observed in MT-treated flowers in the current study could be explained by the effect of MT on convoying appropriate water relations that resulted in maintaining higher levels of RWC than untreated flowers. It is well known that cut roses are susceptible to impaired water balance and therefore keeping water relation is crucial to vase life extension [34]. Increasing the vase life by maintaining the proper water relation and RWC was also observed in gladiolus cut spikes [13] and cut roses [1]. Similarly, Lezoul et al. [30] showed that MT treatment resulted in water relation preservation during postharvest life in cut carnation. Furthermore, MT-induced stomatal closure of cut roses may participate in reducing water loss and consequently maintaining the water balance. Inducing stomatal closure has found to be effective in water balance maintenance in cut roses [1]. It is important to mention that MT role in stomata closure which indirectly contributes to the expanded vase life of ‘First red’ cut rose is new. 

In the current study, the reduction in MDA content due to MT treatment clearly points to minimizing lipid peroxidation in treated flowers which results in membrane integrity maintenance. Decreasing the lipid peroxidation and therefore maintaining MSI has been previously reported in cut flowers [13,35,36]. Our results agreed with those of Lezoul et al. [30] who indicate MT role in retaining the MSI, which contributes to the prolonged vase life of cut carnation. MT-induced reduction in H_2_O_2_ level found in this study is consistent with the observed decline in MDA level of rose flowers, indicative of oxidative stress detoxification. Contrary, untreated rose flowers showed elevated levels of both MDA and H_2_O_2_ resulting in the flower deterioration. Other published works [36,37] similarly report that exposing cut flowers to oxidative injury induces cellular adverse effects and eventually flower senescence. Accordingly, we speculate that membrane integrity retention by MT application relative to untreated flowers most probably related to membrane unsaturated to saturated fatty acids ratio maintenance, which has been reported to be impaired by ROS in peony flowers [38]. 

The oxidative damage obviously impacts the vase life of cut flowers, and thus promoting the antioxidant machinery has been documented to mitigate this damage in various studies [1,13,39]. In support, antioxidant defense systems (non-enzymatic and enzymatic) have been illustrated to defend the cells against the hazardous impacts of oxidative injury and to participate in osmotic adjustment [40,41]. This study also showed that elevated total phenols and GSH levels in response to MT treatment may contribute to lipid peroxidation decrease and hence retaining membrane function; the impact that may associate with senescence regulation of cut rose. Our proposal is consistent with the finding that rose flower senescence was linked to membrane disruption induced by its lipid peroxidation [1], supportive of total phenols and GSH roles in mitigation of oxidative stress adverse effects. Increased total phenols due to external MT supply observed in the current study agrees with the finding reported by Lezoul et al. [30] in carnation cut flowers. Mohammadi et al. [39] demonstrated similarly that phenolic compounds protect lipid membrane oxidation against ROS adverse effects. Gan et al. [42] also indicate that phenols and GSH have non-enzymatic antioxidative functions that contribute to MDA and H_2_O_2_ reduction under MT supplication. On the other hand, it seems that total phenols and GSH generated in non-treated rose flowers were not high enough to provide protection against oxidative damage induced in the cut rose. It is worth reporting that the role of MT in stimulating GSH accumulation in cut *Rosa hybrida* cv. ‘First Red’ is novel. The higher total phenols in MT supplied flowers probably ascribed to polyphenol oxidase activity decline as shown in anthurium flowers [29]. In agreement, Lezoul et al. [30] report that MT treatment delayed carnation polyphenol degradation and maintained total phenol content for a longer period. 

MT treatment increased both non-enzymatic and enzymatic antioxidants in cut roses. Enhanced activities of CAT, APX and GR as well as phenolic compounds and GSH in MT-treated flowers is apparently pointing to their implication in oxidative stress alleviation which retards flower senescence in cut roses. These effectual antioxidant defense systems obviously inhibit the hazardous impacts of oxidative injury and extend vase life of cut roses. Improving the activities of antioxidant enzymes refers to their effective functions in cellular defense mechanism versus oxidative damage under several environmental stresses [43,44,45]. Moreover, enhancing the antioxidant enzyme activity has been previously found to decrease MDA level as one facet of oxidative injury [9].

In this study, the scavenging capacity of MT-treated flowers was enhanced and the IC_50_ values in treated rose flowers were much lower than those recorded by the control. Increasing the radical scavenging potential due to MT treatment in cut roses may be attributed to enhanced levels of both non-enzymatic (phenolics and GSH) and enzymatic antioxidants (CAT, APX and GR). This result is another facet of oxidative damage scavenging induced by MT. Really, this is the first investigation reporting the efficacy of MT on the scavenging activity in “First Red” cut rose. These observations are consistent with the report of Arnao and Hern’andez-Ruiz [18] who illustrated that MT by itself has radical scavenge activity and also stimulates the antioxidant enzyme system in plant tissues reducing the oxidative enzymes activity. In this context, MT treatment has found to play an effective role in enhancing the antioxidant capacity, maintaining redox homeostasis, and therefore, modulating reparation of oxidatively injured proteins in cut anthurium flowers [29]. This observed effect of MT probably responsible for retaining higher MSI in MT-treated rose flowers compared to the control. Enhancing the antioxidant capacity due to MT-treatment in this study is in accordance with the previous report on carnation cut flowers [30]. Therefore, higher RWC, antioxidant contents and enzyme activities under limited water uptake, which is directly associated with MT treatment, consequently the vase life of MT-treated flowers was extended.

## 4. Materials and Methods

### 4.1. Flower Preparation and MT Application

The cut flowers of *Rosa hybrida* cv. ‘First Red’ were obtained from a local grower and immediately transported to the laboratory with stems immersed in tap water-filled buckets. Upon arrival, flowers were cut to 40 cm length and leaves were removed, except the top two leaves. Melatonin (Sigma-Aldrich, St. Louis, MO, USA) was used to prepare the concentrations of 0, 0.1, 0.2 and 0.3 mM using 0.5 mL ethanol and then diluted in 500 mL distilled water. Then, the flowering stems were pulsed in MT concentrations for 30 min and distilled water containing the same ethanol volume was used as a control. During pulsing treatment, the flowers were immersed to a specific height of 5 cm for all treatments. Flowers were then transferred to 500 mL flasks contained distilled water for vase life evaluation. Throughout the vase life period, when necessary, distilled water was added. The experimental design was in a complete randomized system (CRD) of four treatments. Each treatment includes three replicates, five flowers each.

### 4.2. Vase Life 

Flowers life was daily assessed at 20 ± 1 °C, 70 ± 5% RH, and 12 h photoperiod with photosynthetic photon flux density of 20-22 µmol m^−2^ s^−1^. The period from the treatment beginning until the bent neck occurrence or wilting 50% of petals was defined as the vase life [1]. The physiological and biochemical characteristics of the flowers were evaluated at 0, 2, 4, 6, 8 and 10 days of vase life.

### 4.3. Relative Water Content (RWC) 

To measure RWC in flowers, petals from the second outer whorls were used and the following formula reported by Weatherley [46] was used:

(W_fresh_ − W_dry_)/(W_turgid_ − W_dry_) × 100

where W_fresh_ is the sample fresh weight, W_dry_ is the sample dry weight after forty eight hours from oven discation at 70 °C, and W_turgid_ is the sample turgid weight after saturation with distilled water at 4 °C for 24 h. 

### 4.4. Scanning Electron Microscopy (SEM) 

To investigate the stomata observed in lower epidermis of rose leaf, SEM investigation was applied. Leaf segments (∼2 mm × 4 mm) were collected from the second leaf on the 6th day of vase life for both treated and nontreated flowers, and fixed in glutaraldehyde (4%) and phosphate buffer (pH = 6.8) for three days [47]. Segments were then aspirated and dehydrated using a gradually increased concentration of ethanol. Accordingly, segments were dried based on CO_2_ critical point, coated with gold, and then examined at 20 kV using SEM, model JSM-6390LA (JEOL, Tokyo, Japan), followed by photography. 

### 4.5. Total Phenol Content

Total phenol was determined using the methodology of McDonald et al. [48]. A petal sample from the second outer whorls of 0.5 g was stirred with methanol (50 mL) for two days, and the extract was kept at 4 °C. The extract was then diluted (0.5 mL of 0.1 kg L^−1^) and blended with Folin-Ciocalteu reagent (5 mL, 1:10) and 1 M aqueous sodium carbonate (4 mL). Total phenol content was then assessed using a spectrophotometer (Cole-Parmer Ltd., Stone, Staffs, UK, ST15 0SA Model 7205) at 765 nm, and values were expressed in g GAE kg^−1^ DW.

### 4.6. Glutathione (GSH) Determination 

To measure the GSH concentration in petal sample from the second outer whorls, the spectrophotometry method reported by Anderson [49] and slightly modified by Sahoo et al. [50] was used in which the calibration curve of pure GSH as a standard was applied following the linear regression analysis. 

### 4.7. Antioxidant Enzymes 

The activity of catalase (CAT) [EC 1.11.1.6] was assessed using Chandlee and Scandalios’ method [51]. A petal sample (0.5 g) from the second outer whorls was homogenized in 5 mL of 50 mM sodium phosphate buffer (pH 7.5) containing in 1 mM phenylmethylsulfonyl fluoride (PMSF). The extract was then centrifuged at 4 °C for 20 min at 12,000× *g*. The resulting supernatant was used to assay the enzyme. The enzyme extract (0.04 mL) was mixed with H_2_O_2_ (0.4 mL, 15 mM) and potassium phosphate buffer (2.6 mL, 50 mM, pH 7.0). The decomposition of H_2_O_2_ was evaluated by monitoring the absorbance reduction at 240 nm, and CAT activity was recorded (U·mg^−1^ protein), where 1 U = the decline of 1 mM H_2_O_2_·min^−1^·mg^−1^ protein.

Ascorbate peroxidase (APX) [*EC* 1.11.1.11] was also assessed using the protocol of Nakano and Asada [52]. Leaf sample (0.1 g) was ground with 0.2 mL extraction buffer composed of EDTA (3.0 mM), Triton X-100 (1%), Na-phosphate (0.1 M, pH 7.0), polyvinylpyrrolidone [PVP] (1%). The mixture was then centrifuged (10,000× *g*) for 20 min. The absorbance was evaluated at 290 nm to assess APX activity. The reaction buffer, composed of 0.1 mM EDTA, 0.1 mM H_2_O_2_, 0.5 mM ascorbate, and 0.05 mL enzyme extract, was prepared and the reaction was performed at 25 °C for 5 min. APX activity was eventually calculated using the coefficient of absorbance (2.8 mM^−1^·cm^−1^). One unit of APX enzyme can decompose 1.0 µmol of ascorbate per minute.

Glutathione reductase (GR) [EC 1.6.4.2] was evaluated per the protocol of Foyer and Halliwell [53] that was modified by Rao [54]. A leaf sample (0.5 g) was milled with 2.0 mL of the extraction buffer, composed of 3.0 mM EDTA (0.1% PVP), 1 M Na-phosphate (pH 7), and 1.0% Triton X-100. The mixture was then centrifuged (10,000× *g*) for 10 min. The supernatant was assayed for GR activity at 340 nm, following the oxidation of NADPH glutathione-dependent. The reaction mixture was consisted of 0.05 mL of enzyme extract, 0.5 mM glutathione disulfide and 0.2 NADPH, and was kept at 25 °C for 5 min. The correction was applied in the absence of NADPH to overcome the oxidation of glutathione disulfide. The GR activity was eventually measured using the absorbance coefficient of 6.2 mM^−1^·cm^−1^, where one unit of GR was can decompose 1.0 µmol NADPH per minute.

### 4.8. Radical Scavenging Activity (DPPH Assay)

The methodology of Brand-Williams et al. [55] was used for the determination of the scavenging activity of free radicals. A petal sample (0.2 g) from the second outer whorls was weighed out, and 200 mL of methanol was added. It was left at room temperature for 24 h in a shaker to acerate. Then, the sample was filtered (Whatman No. 1). To remove the methanol, evaporation at in a fume hood room temperature was done. The resulting extract was kept for later analysis. The 1.1-diphenyl-2-picryl-hydrazil (DPPH) reagent was used for this assay. Several concentrations of flower extract viz. 1, 2, 3 and 4 µg·mL^−1^ were dissolved in aqueous methanol (85%). About 0.5 mL of the extract was added to 1.5 mL methanolic solution of DPPH (20 µg·mL^−1^), and stirred well. Thirty minutes after the reaction, the decolorizing processes was assessed and compared with the blank at 517 nm. The DPPH activity was determined as a percentage of inhibition (I%), as follow:

I (%) = 100 × (A_blank_ − A_sample_)/A_blank_

where A_sample_ and A_blank_ are the absorbances of the sample and the blank after 30 min of the reaction, respectively. The extract sample that generates 50% inhibition was considered IC_50_ (the activity of antiradical), and was presented in mmol·kg^−1^ FW.

### 4.9. Hydrogen Peroxide (H_2_O_2_) Assessment

The generation of H_2_O_2_ in petal samples from the second outer whorls was also determined [56]. Flower sample (0.5 g) was homogenized with 6 mL chilled acetone (100%), and the mixture was centrifuged (12,000× *g*) at 4 °C for 10 min. A 1 mL sample of the extract was added to 0.1 mL Ti(SO_4_)^2^ (5%) and 0.2 mL NH_4_OH (concentrated solution), and centrifugated at 3000× *g* for 10 min. The pellets were then dissolved in 4 mL H_2_SO_4_ (2 M), and the absorbance of titanium-peroxide complex was then assessed at 412 nm. The absorbance was calibrated to a standard curve following known H_2_O_2_ levels, and H_2_O_2_ content was presented in mmol·kg^−1^ FW.

### 4.10. Assessment of Lipid Peroxidation 

The content of MDA was used to assess lipid peroxidation, [57]. A petal sample from the second outer whorls (0.2 g) was homogenized in 2 mL trichloroacetic acid (0.1%) and centrifuged (14,000× *g*) for 15 min. An aliquot sample (2 mL) was added to 3 mL thiobarbituric acid (0.5%) and trichloroacetic acid (5%), and kept for 30 min. The mixture was then cooled in ice, and centrifuged (5000× *g*) for 15 min. MDA content (μmol mL^−1^) was calculated using the following equation:

MDA content = 6.45 × (A_532_ − A_600_) − 0.56 × A_450_,

where A is the supernatant’s optical density at 450, 532, and 600 nm.

### 4.11. Membrane Stability Index (MSI) 

This was performed as described [58] using two petal samples from the second outer whorls of 0.2 g each in two separate flasks (50 mL) containing 20 mL deionized water. The first flask was kept at 40 °C for 30 min, but the second flask was kept in hot water bath (100 °C) for 15 min. The conductivity of both samples (C_1_ and C_2_) were then assessed using a conductivity meter, and ion leakage was used to determine MSI, as follow:

MSI = [1 − (C_1_/C_2_)] × 100.

### 4.12. Statistical Analysis 

This study was repeated twice during March and April 2021 and data was pooled and SPSS 13.3 program (IBM, New York, NY, USA) was applied to conduct the analysis of variance (ANOVA). Mean separations were performed using Tukey-Kramer’s multiple range test at *p* ≤ 0.05, and the results were presented in means ± SE (*n* = 6). 

## 5. Conclusions

This invistigation is the first to report that MT had the capability to extend the longevity of *Rosa hybrida* cv. ‘First Red’. The impacts of MT on maintaining the quality of cut roses were attributed to improving enzymatic and non-enzymatic antioxidant defense systems that in turn reduced lipid peroxidation, H_2_O_2_ accumulation and maintained membrane function. MT may be recommended as a novel preservative to extend the vase life of cut roses at commercial scale in floral industry.

## Data Availability

The data sets supporting the results of this research are included within the article.

## References

[1] Hassan F.A.S., Mazrou R., Gaber A., Hassan M. (2020). Moringa extract preserved the vase life of cut roses through maintaining water relations and enhancing antioxidant machinery. Postharvest Biol. Technol..

[2] Hassan F.A.S., Schmidt G. (2004). Postharvest characteristics of cut carnations as the result of chemical treatments. Acta Agron. Hung..

[3] Lu P., Cao J., He S., Liu J., Li H., Cheng G., Ding Y., Joyce D.C. (2010). Nano-silver pulse treatments improve water relations of cut rose cv. Movie Star flowers. Postharvest Biol. Technol..

[4] Saeed T., Hassan I., Abbasi N., Jilani G. (2014). Effect of gibberellic acid on the vase life and oxidative activities in senescing cut gladiolus flowers. Plant Growth Regul..

[5] Hatamzadeh A., Hatami M., Ghasemnezhad M. (2012). Efficiency of salicylic acid delay petal senescence and extended quality of cut spikes of *Gladiolus grandiflora* cv ‘wing’s sensation’. Afric. J. Agric. Res..

[6] Hassan F.A.S., Ali E.F. (2014). Protective effects of 1-methylcyclopropene and salicylic acid on senescence regulation of gladiolus cut spikes. Sci. Hortic..

[7] Aalifar M., Aliniaeifard S., Arab M., Mehrjerdi M.Z., Serek M. (2020). Blue light postpones senescence of carnation flowers through regulation of ethylene and abscisic acid pathway-related genes. Plant Physiol. Biochem..

[8] Rashidiani N., Nazari F., Javadi T., Samadi S. (2020). Comparative postharvest responses carnation and chrysanthemum to synthesized silver nanoparticles (Agnps). Adv. Hortic. Sci..

[9] Ali E.F., Issa A.A., Al-Yasi H.M., Hessini K., Hassan F.A.S. (2022). The efficacies of 1-methylcyclopropene and chitosan nanoparticles in preserving the postharvest quality of Damask rose and their underlying biochemical and physiological mechanisms. Biology.

[10] Mittler R. (2002). Oxidative stress, antioxidants and stress tolerance. Trends Plant Sci..

[11] Guan Y., Hu W., Jiang A., Xu Y., Zhao M., Yu J., Ji Y., Sarengaowa, Yang X., Feng K. (2020). The effect of cutting style on the biosynthesis of phenolics and cellular antioxidant capacity in wounded broccoli. Food Res. Int..

[12] Akhtar G., Rajwana I.A., Sajjad Y., Asif M., Muhammad S., Kashif A., Sami R., Hafiz U., Faried N., Farooq A. (2021). Do natural leaf extracts involve regulation at physiological and biochemical levels to extend vase life of gladiolus cut flowers?. Sci. Hortic..

[13] Hassan F.A.S., Fetouh M.I. (2019). Does moringa leaf extract have preservative effect improving the longevity and postharvest quality of gladiolus cut spikes?. Sci. Hortic..

[14] Reiter R.J., Tan D.X., Zhou Z., Cruz M.H., Fuentes-Broto L., Galano A. (2015). Phytomelatonin: Assisting plants to survive and thrive. Molecules.

[15] Fan J., Xie Y., Zhang Z., Chen L. (2018). Melatonin: A multifunctional factor in plants. Int. J. Mol. Sci..

[16] Pang L., Wu Y., Pan Y., Ban Z., Li L., Li X. (2020). Insights into exogenous melatonin associated with phenylalanine metabolism in postharvest strawberry. Postharvest Biol. Technol..

[17] Hernández-Ruiz J., Arnao M.B. (2018). Relationship of melatonin and salicylic acid in biotic/abiotic plant stress responses. Agronomy.

[18] Arnao M.B., Hernández-Ruiz J. (2019). Melatonin: A new plant hormone and/or a plant master regulator?. Trends Plant Sci..

[19] Arnao M.B., Hernández-Ruiz J. (2020). Melatonin in flowering, fruit set and fruit ripening. Plant Reprod..

[20] Sharif R., Xie C., Zhang H., Arnao M.B., Ali M., Ali Q., Muhammad I., Shalmani A., Nawaz M.A., Chen P. (2018). Melatonin and its effects on plant systems. Molecules.

[21] Zhang N., Sun Q., Li H., Li X., Cao Y., Zhang H., Li S., Zhang L., Qi Y., Ren S. (2016). Melatonin improved anthocyanin accumulation by regulating gene expressions and resulted in high reactive oxygen species scavenging capacity in cabbage. Front. Plant Sci..

[22] Debnath B., Islam W., Li M., Sun Y.T., Lu X.C., Mitra S., Hussain M., Liu S., Qiuet D. (2019). Melatonin mediates enhancement of stress tolerance in plants. Int. J. Mol. Sci..

[23] Ma D.Y., Xu Y., Zhang Z.Q., Li B.Q., Chen T., Tian S.P. (2018). Efficacy of ABA- mimicking ligands in controlling water loss and maintaining antioxidative capacity of spinacia oleracea. J. Agric. Food Chem..

[24] Aghdam M.S., Luo Z., Li L., Jannatizadeh A., Fard J.R., Pirzad F. (2020). Melatonin treatment maintains nutraceutical properties of pomegranate fruits during cold storage. Food Chem..

[25] Tan X., Zhao Y., Shan W., Kuang J., Lu W., Su X., Tao N., Lakshmanan P., Chen J. (2020). Melatonin delays leaf senescence of postharvest Chinese flowering cabbage through ROS homeostasis. Food Res. Int..

[26] Deng B., Xia C., Tian S., Shi H. (2021). Melatonin reduces pesticide residue, delays senescence, and improves antioxidant nutrient accumulation in postharvest jujube fruit. Postharvest Biol. Technol..

[27] Rastegar S., Khankahdani H.H., Rahimzadeh M. (2020). Effects of melatonin treatment on the biochemical changes and antienzyme activity of mango fruit during storage. Sci. Hortic..

[28] Onik J.C., Wai S.C., Li A., Lin Q., Sun Q., Wang Z., Duan Y. (2021). Melatonin treatment reduces ethylene production and maintains fruit quality in apple during postharvest storage. Food Chem..

[29] Aghdam M.S., Jannatizadeh A., Nojadeh M.S., Ebrahimzadeh A. (2019). Exogenous melatonin ameliorates chilling injury in cut anthurium flowers during low temperature storage. Postharvest Biol. Technol..

[30] Lezoul N.E., Serrano M., Ruiz-Aracil M.C., Belkadi M., Castillo S., Valero D., Guill´en F. (2022). Melatonin as a new postharvest treatment for increasing cut carnation (*Dianthus caryophyllus* L.) vase life. Postharvest Biol. Technol..

[31] Hassan F., Ali E.F., Mahfouz S. (2012). Comparison between different fertilization sources, irrigation frequency and their combinations on the growth and yield of coriander plant. Aust. J. Basic Appl. Sci..

[32] Kahil A.A., Hassan F.A.S., Ali E.F. (2017). Influence of bio-fertilizers on growth, yield and anthocyanin content of *Hibiscus sabdariffa* L. plant under Taif region conditions. Annu. Res. Rev. Biol..

[33] Zhao D., Wang R., Meng J., Zhiyuan L., Wu Y., Tao J. (2017). Ameliorative effects of melatonin on dark-induced leaf senescence in gardenia (*Gardenia jasminoides* Ellis): Leaf morphology, anatomy, physiology and transcriptome. Sci. Rep..

[34] Rafi Z.N., Ramezanian A. (2013). Vase life of cut rose cultivars ‘Avalanche’ and ‘Fiesta’ as affected by Nano-Silver and S-carvone treatments. S. Afr. J. Bot..

[35] Latif H.H., Mohamed H.I. (2016). Exogenous applications of moringa leaf extract effect on retro transposon, ultra-structural and biochemical contents of common bean plants under environmental stresses. S. Afr. J. Bot..

[36] Hassan F., Ali E., Mazrou R. (2020). Involvement of ethylene synthetic inhibitors in regulating the senescence of cut carnations through membrane integrity maintenance. J. Hortic. Res..

[37] Hassan F.A.S., Ali E.F., El-Deeb B. (2014). Improvement of postharvest quality of cut rose cv. ‘First Red’ by biologically synthesized silver nanoparticles. Sci. Hortic..

[38] Wang S., Xue J., Zhang S., Zheng S., Xue Y., Xu D., Zhang X. (2020). Composition of peony petal fatty acids and flavonoids and their effect on Caenorhabditis elegans lifespan. Plant Physiol. Biochem..

[39] Mohammadi M., Aelaei M., Saidi M. (2020). Pre-harvest and pulse treatments of spermine, γ-and β-aminobutyric acid increased antioxidant activities and extendedthe vase life of gerbera cut flowers ‘Stanza’. Ornam. Hortic..

[40] Mansour M.M.F., Ali E.F. (2017). Evaluation of proline functions in saline conditions. Phytochemistry.

[41] Ali E.F., El-Shehawi A.M., Ibrahim O.H.M., Abdul-Hafeez E.Y., Moussa M.M., Hassan F.A.S. (2021). A vital role of chitosan nanoparticles in improvisation the drought stress tolerance in *Catharanthus roseus* (L.) through biochemical and gene expression modulation. Plant Physiol. Biochem..

[42] Gan J., Feng Y., He Z., Li H., Zhang H. (2017). Correlations between antioxidant activity and alkaloids and phenols of maca (*Lepidium meyenii*). J. Food Qual..

[43] Attia H., Al-Yasi H., Alamer K., Ali E., Hassan F., El-shazly S., Hessini K. (2020). Induced anti-oxidation efficiency and others by salt stress in *Rosa damascena* Miller. Sci. Hortic..

[44] Hessini K., Wasli H., Al-Yasi H.M., Ali E.F., Issa A.A., Hassan F.A.S., Siddique K.H.M. (2022). Graded moisture deficit effect on secondary metabolites, antioxidant, and inhibitory enzyme activities in leaf extracts of *Rosa damascena* Mill. var. trigentipetala. Horticulturae.

[45] Zhou Q., Ma C., Cheng S., Wei B., Liu X., Ji S. (2014). Changes in antioxidative metabolism accompanying pitting development in stored blueberry fruit. Postharvest Biol. Technol..

[46] Weatherley P.E. (1950). Studies in the water relations of the cotton plant.1. The field measurements of water deficit in leaves. New Phytol..

[47] Zobayed S.M.A., Armstrong J., Armstrong W. (2001). Leaf anatomy of in vitro tobacco and cauliflower plantlets as affected by different types of ventilation. Plant Sci..

[48] McDonald S., Prenzler P.D., Antolovich M., Robards K. (2001). Phenolic content and antioxidant activity of olive extracts. Food Chem..

[49] Anderson M.E. (1985). Determination of glutathione and glutathione disulfide in biological samples. Methods Enzymol..

[50] Sahoo S., Awasthi J.P., Sunkar R., Panda S.K., Sunkar R. (2017). Determining glutathione levels in plants. Plant Stress Tolerance.

[51] Chandlee J.M., Scandalios J.G. (1984). Analysis of variants affecting the catalase developmental program in maize scutellum. Theor. Appl. Genet..

[52] Nakano Y., Asada K. (1981). Hydrogen peroxide is scavenged by ascorbate-specific peroxidase in spinach chloroplasts. Plant Cell Physiol..

[53] Foyer C.H., Halliwell B. (1976). The presence of glutathione and glutathione reductase in chloroplasts: A proposed role in ascorbic acid metabolism. Planta.

[54] Rao M.V. (1992). Cellular detoxifying mechanisms determine the age dependent injury in tropical trees exposed to SO_2_. J. Plant Physiol..

[55] Brand-Williams W., Cuvelier M.E., Berset C. (1995). Use of a free radical method to evaluate antioxidant activity. Lebensm. Wiss. Technol..

[56] Patterson B.D., Macrae E.A., Ferguson I.B. (1984). Estimation of hydrogen peroxide in plant extracts using titanium (IV). Anal. Chem..

[57] Hodges D.M., Delong J.M., Forney C.F., Prange R.K. (1999). Improving the thiobarbituric acidreactive-substances assay for estimating lipid peroxidation in plant tissue containing anthocyanin and other interfering compounds. Planta.

[58] Sairam R.K., Deshmukh P.S., Shukla D.S. (1997). Tolerance to drought and temperature stress in relation to increased antioxidant enzyme activity in wheat. J. Agron. Crop Sci..

